# Supplementary material for: Electroactive Ultra-Thin rGO-Enriched FeMoO4 Nanotubes and MnO2 Nanorods as Electrodes for High-Performance All-Solid-State Asymmetric Supercapacitors
Source: Nanomaterials (Basel). 2020 Feb 9;10(2):289. doi: 10.3390/nano10020289 (PMC7075176; doi:10.3390/nano10020289)
Supplement: Supplementary file 1 [file nanomaterials-10-00289-s001.pdf]

nanomaterials

Supporting Information

Electroactive Ultra-Thin rGO-Enriched $FeMoO_4$ Nanotubes and MnO_2 Nanorods as Electrodes for High-Performance All-Solid-State Asymmetric Supercapacitors

Kugalur Shanmugam Ranjith [1], Ganji Seeta Rama Raju [1], Nilesh R. Chodankar [1], Seyed Majid Ghoreishian [2], Cheol Hwan Kwak [2], Yun Suk Huh [2,*] and Young-Kyu Han [1,*]

[1] Department of Energy and Material Engineering, Dongguk University-Seoul, Seoul 04620, Korea; ranjuphy@buc.edu.in (K.S.R.); gseetaramaraju7@dongguk.edu (G.S.R.R.); chodankarnilesh@dongguk.edu (N.R.C.)

[2] Department of Biological Engineering, Inha University, Incheon 22212, Korea; m.ghoreishian.1985@inha.edu (S.M.G.); kwakch@krict.re.kr (C.H.K.)

* Correspondence: yunsuk.huh@inha.ac.kr (Y.S.H.); ykenergy@dongguk.edu (Y.-K.H.)

Received: 10 January 2020; Accepted: 5 February 2020; Published: 9 February 2020

Abstract: A flexible asymmetric supercapacitor (ASC) with high electrochemical performance was constructed using reduced graphene oxide (rGO)-wrapped redox-active metal oxide-based negative and positive electrodes. Thin layered rGO functionality on the positive and the negative electrode surfaces has promoted the feasible surface-active sites and enhances the electrochemical response with a wide operating voltage window. Herein we report the controlled growth of rGO-wrapped tubular $FeMoO_4$ nanofibers (NFs) via electrospinning followed by surface functionalization as a negative electrode. The tubular structure offers the ultrathin-layer decoration of rGO inside and outside of the tubular walls with uniform wrapping. The rGO-wrapped tubular $FeMoO_4$ NF electrode exhibited a high specific capacitance of 135.2 F g^{-1} in Na_2SO_4 neutral electrolyte with an excellent rate capability and cycling stability (96.45% in 5000 cycles) at high current density. Meanwhile, the hydrothermally synthesized binder-free rGO/MnO_2 nanorods on carbon cloth (rGO-MnO_2@CC) were selected as cathode materials due to their high capacitance and high conductivity. Moreover, the ASC device was fabricated using rGO-wrapped $FeMoO_4$ on carbon cloth (rGO-$FeMoO_4$@CC) as the negative electrode and rGO-MnO_2@CC as the positive electrode (rGO-$FeMoO_4$@CC/rGO-MnO_2@CC). The rationally designed ASC device delivered an excellent energy density of 38.8 W h kg^{-1} with a wide operating voltage window of 0.0–1.8 V. The hybrid ASC showed excellent cycling stability of 93.37% capacitance retention for 5000 cycles. Thus, the developed rGO-wrapped $FeMoO_4$ nanotubes and MnO_2 nanorods are promising hybrid electrode materials for the development of wide-potential ASCs with high energy and power density.

Keywords: electrospinning; $FeMoO_4$ nanotubes; rGO wrapping; MnO_2-rGO; asymmetric supercapacitors

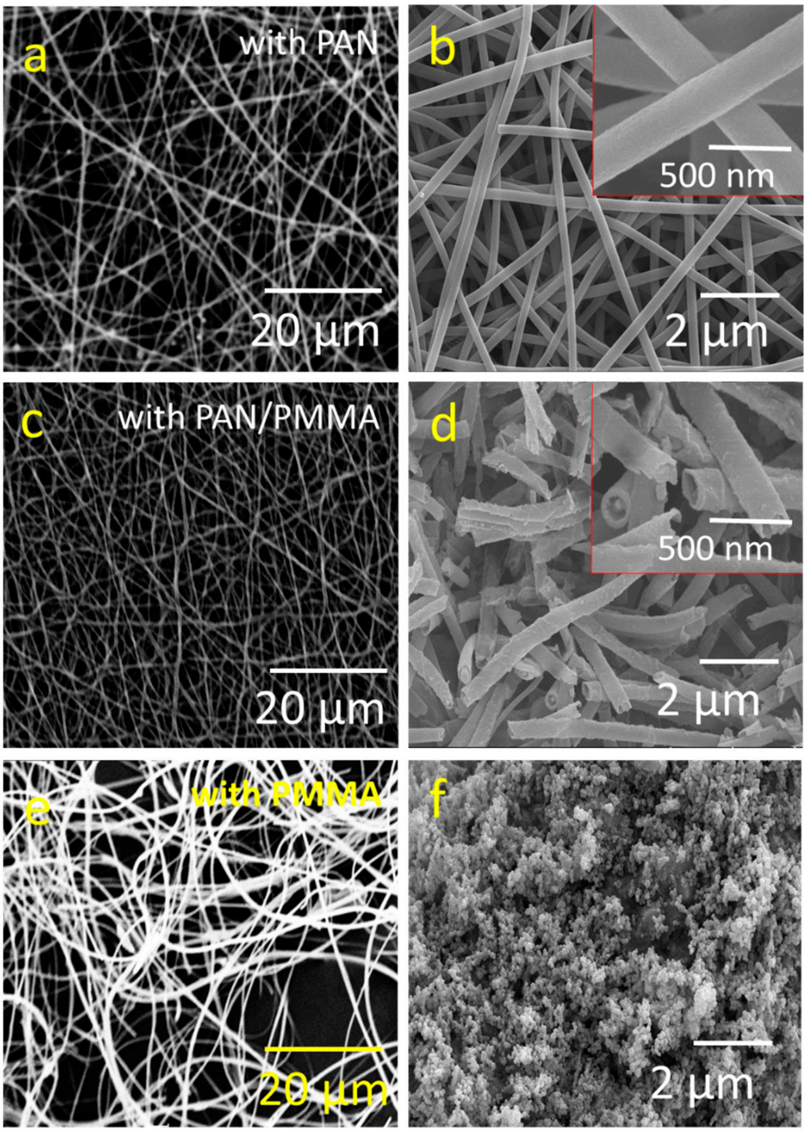

Figure S1. SEM image of the as-spun (a, c, e) and annealed (b, d, f) nanofibers with different polymer precursors. (a, b) FeMo-PAN, (c, d) FeMo-PAN/PMMA and (e, f) FeMo-PMMA.

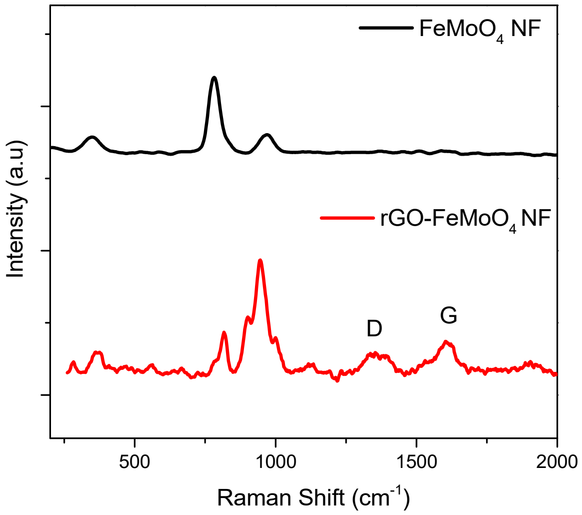

Figure S2. Raman analysis of the pristine and rGO wrapped $FeMoO_4$ tubular nanofibers.

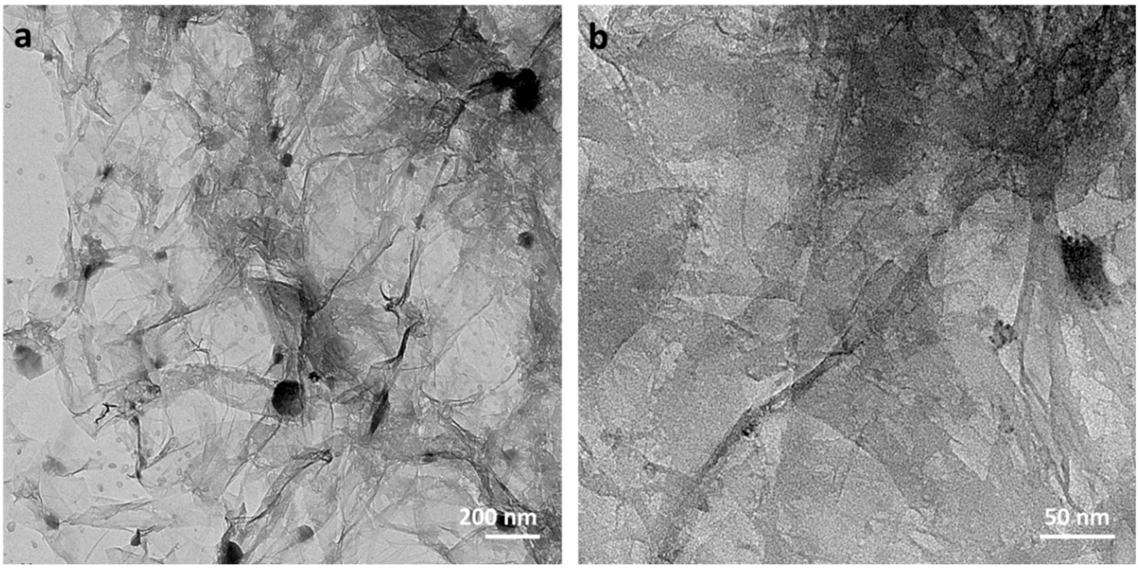

Figure S3. TEM images of the ultra-thin rGO nanoflakes prepared throught the thermal reduction of GO.

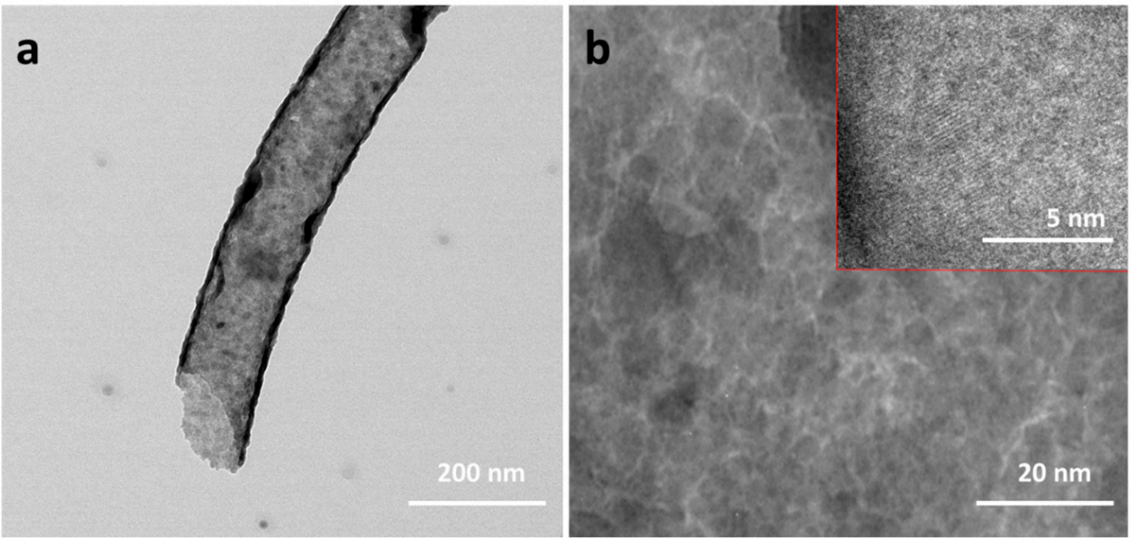

Figure S4. TEM images of the pristine $FeMoO_4$ tubular nanofibers, inset shows the HRTEM images of the respective tubular nanofiber.

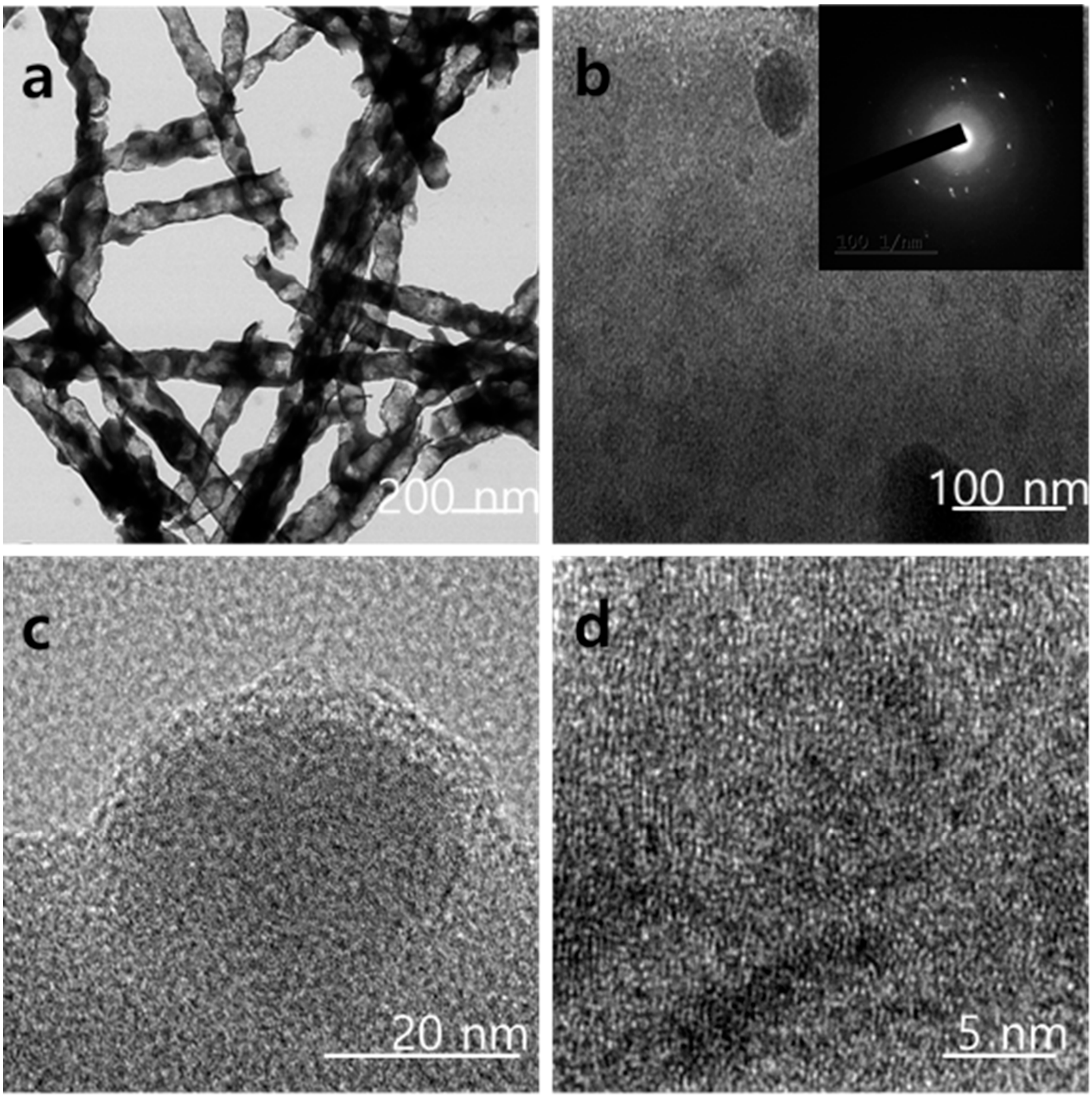

Figure S5. TEM images of the pristine rGO wrapped $FeMoO_4$ tubular nanofibers, inset shows the SEAD pattern of the respective tubular nanofiber. .

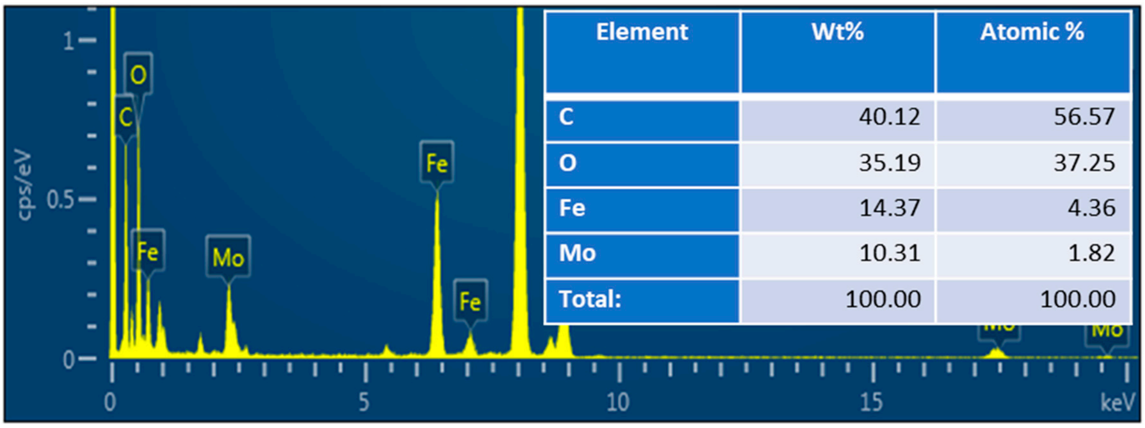

Element	Wt%	Atomic %
C	40.12	56.57
O	35.19	37.25
Fe	14.37	4.36
Mo	10.31	1.82
Total:	100.00	100.00

Figure S6. EDAX results of the rGO wrapped $FeMoO_4$ nanofibers.

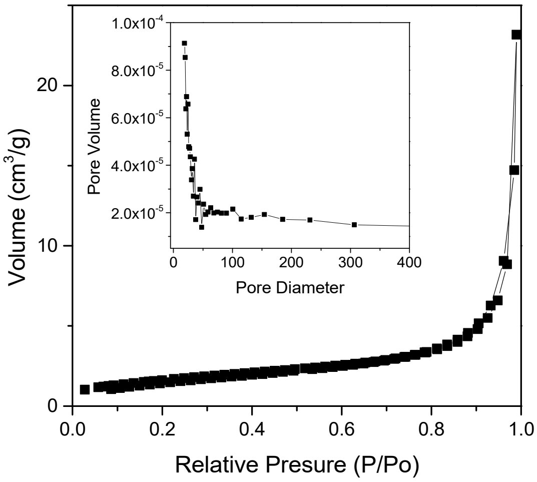

Figure S7. N_2 adsorption-desorption isotherm of rGO wrapped $FeMoO_4$ nanofibers. Inset shows the corresponding pore size distribution curve of the tubular nanofibers.

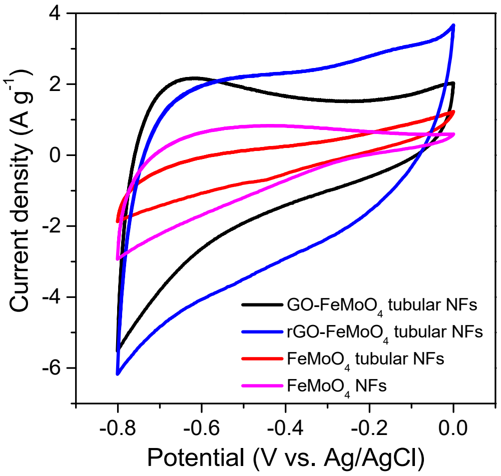

Figure S8. CV curve of the $FeMoO_4$, and rGO-$FeMoO_4$ electrodes at 10 mV s^{-1} in a three-electrode cell.

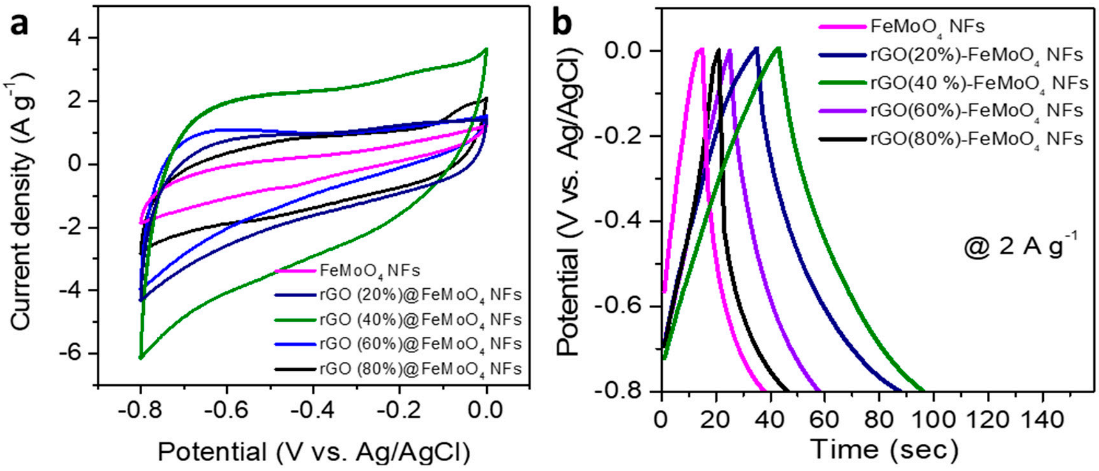

Figure S9. CV and GCD curve of the $FeMoO_4$ with different loading density of rGO (20, 40, 60 % and 80 %).

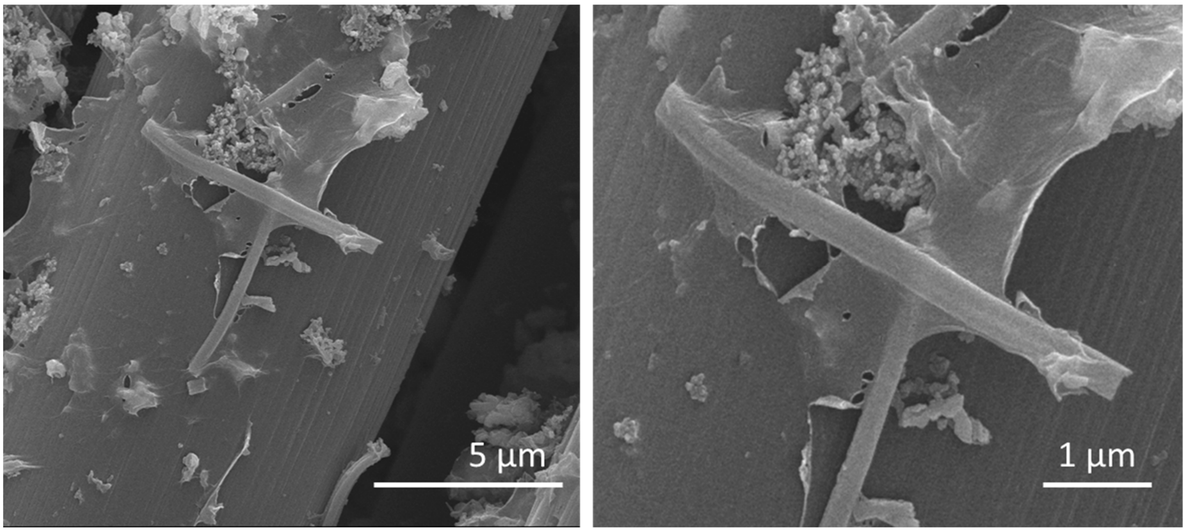

Figure S10. SEM image of the rGO-$FeMoO_4$@CC electrode after electrochemical cyclic performances.

Table S1. Comparison of the energy density of the rGO-MnO_2//rGO-$FeMoO_4$ ASC device with previously reported MnO_2 based and other ASC systems.

ASC device	Voltage (V)	E (W h kg^{-1})	P (W kg^{-1})	Ref.
$ZnCo_2O_4$–MnO_2//AC	1.6	69	867	[1]
$ZnCo_2O_4$/NG//AC	1.6	28.3	500	[2]
$ZnCo_2O_4$@$Ni_xCo_{2x}(OH)_{6x}$//AC	1.7	26.2	511.8	[3]
Co_3O_4@MnO_2//MEGO	1.6	17.7	158	[4]
$ZnCo_2O_4$@MnO_2//AC	1.5	29.41	628.42	[5]
$ZnCo_2O_4$@$ZnWO_4$//AC	1.6	24	400	[6]
MnO_2//Fe_2O_3	1.6	0.55		[7]
$CaMoO_4$//AC	1.6	18.7	362	[8]
MnO_2//Fe_2O_3	1.8	53.55	1280	[9]
MnO_2-GNS//FeOOH-GNS-CNTs	1.7	30.4	237.6	[10]
CNT@NiO//CNT@Fe_2O_3	1.6	63.3	1600	[11]
MnO_2-MWCNT//VN-MWCNT	1.8	38.7	730	[12]
GNR/MnO_2//GNR	2.0	29.4	12.1	[13]
MnO_2//FeOOH	1.85	24	450	[14]
MnO_2 nanowire//graphene	2	30.4	100	[15]
rGO-MnO_2//rGO-$FeMnO_4$*	2.2	31.8	1099	This work*

References

1. V. S. Kumbhar, D. H. Kim, Hierarchical coating of MnO_2 nanosheets on $ZnCo_2O_4$ nanoflakes for enhanced electrochemical performance of asymmetric supercapacitors, *Electrochim. Acta*, 271 (2018) 284-296.
2. X. W. Ma, P. Zhang, Y. Y. Zhao, Y. Liu, J. Li, J. Y. Zhou, X. J. Pan, E. Q. Xie, Role of N doping on the electrochemical performances of $ZnCo_2O_4$ quantum dots/reduced graphene oxide composite nanosheets, *Chem. Eng. J.*, 327 (2017) 1000-1010.
3. W. Fu, Y. Wang, W. Han, Z. Zhang, H. Zha, and E. Xie, Construction of hierarchical $ZnCo_2O_4$@$Ni_xCo_{2x}(OH)_{6x}$ core/shell nanowire arrays for high-performance supercapacitors, *J. Mater. Chem. A*, 4 (2016) 173-182.
4. M. Huang, Y. Zhang, F. Li, L. Zhang, Z. Wen, Q. Liu, Facile synthesis of hierarchical Co_3O_4@MnO_2 core-shell arrays on Ni foam for asymmetric supercapacitors, *J. Power Sources*, 252 (2014) 98-106.
5. D. Yu, Z. Zhang, Y. Meng, Y. Teng, Y. Wu, X. Zhang, Q. Sun, W. Tong, X. Zhao, and X. Liu, The synthesis of hierarchical $ZnCo_2O_4$@MnO_2 core-shell nanosheet arrays on Ni foam for high-performance all-solid-state asymmetric supercapacitors, *Inorg. Chem. Front.*, 5 (2018) 597-604.

6. L. Xie, Y. Liu, H. Bai, C. Li, B. Mao, L. Sun, W. Shi, Core-shell structured $ZnCo_2O_4$@$ZnWO_4$ nanowire arrays on nickel foam for advanced asymmetric supercapacitors. *J. Colloid Interface Sci*., 531 (2018) 64-73.
7. P. Yang, Y. Ding, Z. Lin, Z. Chen, Y. Li, P. Qiang, M. Ebrahimi, W. Mai, C. P. Wong, and Z. L. Wang, Low-cost high performance solid-state asymmetric supercapacitors based on MnO_2 nanowires and Fe_2O_3 nanotubes, Nano Lett. 14 (2014) 731–736.
8. J. Bhagwan, S. K. Hussain, J. S. Yu. Facile Hydrothermal Synthesis and Electrochemical Properties of $CaMoO_4$ Nanoparticles for Aqueous Asymmetric Supercapacitors. *ACS Sustainable Chem. Eng*., 7 (2019) 12340-12350.
9. W. Liu, M. Zhu, J. Liu, X. Li, J. Liu, Flexible asymmetric supercapacitor with high energy density based on optimized MnO_2 cathode and Fe_2O_3 anode, *Chinese Chemical Letters*, 30 (2019) 750-756.
10. C. Long, L. Jiang, T. Wei, J. Yan, and Z. Fan, High-performance asymmetric supercapacitors with lithium intercalation reaction using metal oxide-based composites as electrode materials, *J. Mater. Chem. A*, 2 (2014) 16678-16686.
11. S. Zhang, X. Wang, Y. Li, X. Mu, Y. Zhang, J. Du, G. Liu, X. Hua, Y. Sheng, E. Xie, and Z. Zhang, Facile synthesis of carbon nanotube-supported NiO//Fe_2O_3 for all-solid-state supercapacitors, *Beilstein J. Nanotechnol*., 10 (2019) 1923-1932.
12. X. Y. Yu, H. Hu, Y. Wang, H. Chen, X. W. Lou, Ultrathin MoS_2 Nanosheets Supported on N-doped Carbon Nanoboxes with Enhanced Lithium Storage and Electrocatalytic Properties, *Angew. Chem., Int. Ed*., 54 (2015) 7395-7398.
13. Z. Wu, B. Li, Y. Xue, J. Li, Y. Zhang and F. Gao, Fabrication of defect-rich MoS_2 ultrathin nanosheets for application in lithium-ion batteries and supercapacitors J. Mater. Chem. A, 3 (2015) 19445–19454.
14. B. Xie, Y. Chen, M. Yu, T. Sun, L. Lu, T. Xie, Y. Zhang, Y. Wu, Hydrothermal synthesis of layered molybdenum sulfide/N-doped graphene hybrid with enhanced supercapacitor performance, *Carbon*, 99 (2016) 35-42.
15. M. Kim, Y. Hwang, J. Kim, Graphene/MnO_2-based composites reduced via different chemical agents for supercapacitors, J. Power Sources, 239 (2013) 225-233.
